# Synthesis of Silver Nanoparticles Using Buchu Plant Extracts and Their Analgesic Properties

**DOI:** 10.3390/molecules21060774

**Published:** 2016-06-14

**Authors:** Herbert Chiguvare, Opeoluwa O. Oyedeji, Reuben Matewu, Olukayode Aremu, Idris A. Oyemitan, Adebola O. Oyedeji, Benedicta N. Nkeh-Chungag, Sandile P. Songca, Sneha Mohan, Oluwatobi S. Oluwafemi

**Affiliations:** 1 Department of Chemistry, Faculty of Science and Agriculture, University of Fort Hare, Private Bag X1314, Alice 5700, South Africa; cherbert.jr@gmail.com; 2 1124 Buchanan Street, Ginsburg, King William’s Town 5601, Eastern Cape, South Africa; reubenmatewu@gmail.com; 3 Department of Physiology, Faculty of Health Sciences, Walter Sisulu University, Nelson Mandela Drive Campus, Mthatha 5117, South Africa; snehakoottungal@yahoo.co.in; 4 Department of Chemical and Physical Sciences, Faculty of Natural Sciences, Walter Sisulu University, Nelson Mandela Drive Campus, P. Bag X1, Mthatha 5117, South Africa; oyemix@yahoo.com (I.A.O.); aoyedeji@wsu.ac.za (A.O.O.); spsongca@wsu.ac.za (S.P.S.); 5 Department of Biological and Environmental Science Chemistry, Faculty of Natural Science, Walter Sisulu University, Nelson Mandela Drive Campus, P. BagX1, Mthatha 5117, South Africa; bnkehchungag@wsu.ac.za; 6 Centre for Nanomaterials Science Research, University of Johannesburg, Johannesburg Doornfontein 2028, South Africa; snehamkoottungal@gmail.com; 7 Department of Applied Chemistry, University of Johannesburg, Doornfontein Campus, P.O. Box 17011, Doornfontein 2028, Gauteng, South Africa

**Keywords:** silver nanoparticles, analgesic activity, phytochemicals, buchu

## Abstract

We herein report for the first time the synthesis and analgesic properties of silver nanoparticles (Ag-NPs) using buchu plant extract. The as-synthesised Ag-NPs at different temperatures were characterised by UV-Vis spectroscopy, Fourier transform infra-red spectroscopy (FTIR) and transmission transform microscopy (TEM) to confirm the formation of silver nanoparticles. Phytochemical screening of the ethanolic extract revealed the presence of glycosides, proteins, tannins, alkaloids, flavonoids and saponins. The absorption spectra showed that the synthesis is temperature and time dependent. The TEM analysis showed that the as-synthesised Ag-NPs are polydispersed and spherical in shape with average particle diameter of 19.95 ± 7.76 nm while the FTIR results confirmed the reduction and capping of the as-synthesised Ag-NPs by the phytochemicals present in the ethanolic extract. The analgesic study indicated that the combined effect of the plant extract and Ag-NPs is more effective in pain management than both the aspirin drug and the extract alone.

## 1. Introduction

Synthesis of nanoparticles using plants extract has become one of the most common greener approaches since most plants are biocompatible and are safe to handle [1]. Plant mediated synthesis of silver nanoparticles makes use of the wide variability of functional groups available in the plant which can act as both reducing and capping agents [2,3]. Plant metabolites such as flavonoids and carbohydrate have been reported to act as electron shuttling agents for the reduction of silver ions to silver nanoparticles [3]. The electrostatic interactions and the hydrogen bond network between the silver nanoparticles and the phytochemicals provide stability to the nanoparticle system and control crystal growth [4].

Buchu plant belongs to the species betulina, genus Agathosma and the family Rutaceae [5]. It is native to South Africa and it is a plant well known for its production of essential oil [5]. The essential oil is used in the manufacture of flavourings and perfumes [6]. In traditional medical practice, buchu is most commonly taken orally in aqueous infusion extracts for external application as an antiseptic wash, cleansing of wounds and treating rheumatism [6]. It also has prominent uses as a remedy for indigestion, urinary tract infections and acute inflammation [7]. Thus, it is expected that the use of this plant in the synthesis of Ag-NPs will lead to a synergistic product with high medicinal value.

In view of the excellent properties of buchu plant, we herein report the synthesis of Ag-NPs using this plant and determine the analgesic property of the synergised product. As far as the authors know there are no reports on the synthesis of Ag-NPs using this plant and its analgesic properties. The synthesis was carried out at different temperatures and the as-synthesised Ag-NPs were characterised using UV-Vis spectroscopy, Fourier transform infra-red spectroscopy (FTIR) and transmission transform microscopy (TEM). The analgesic study shows that the combined effect of the plant extract and Ag-NPs is more effective in pain management than the both aspirin drug and the extract alone.

## 2. Results and Discussion

### 2.1. Extraction and Phytochemical Screening

The results from phytochemical screening of the ethanolic extract revealed the presence of glycosides, carbohydrates, flavonoids, alkaloids, steroids, terpenes, tannins, saponins and proteins as shown in Table 1.

### 2.2. UV-Vis Analysis

**Figure 1:**
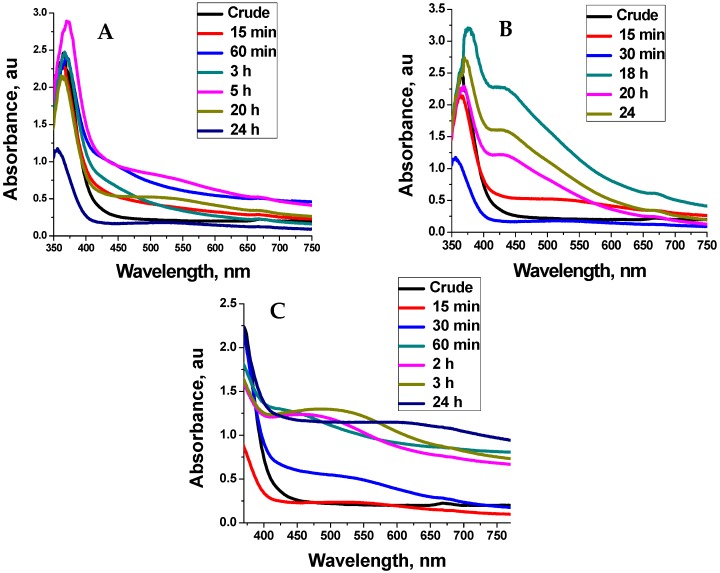
Absorption spectra for the Ag-nanoparticles synthesised at 40 °C (**A**) 60 °C (**B**) and 75 °C (**C**) at different reaction times.

The absorption spectra for the as-synthesised Ag-NPs at different temperature is shown in Figure 1. The absorption spectra showed that the synthesis of silver nanoparticles mediated by buchu ethanolic extract is temperature and reaction time dependent. At 40 °C, broad SPR peaks at 480 nm were observed indicating the presence of polydispersed nanoparticles. An increase in the temperature to 60 °C initially produced SPR peaks at 550 nm. However, as the reaction time increased there was a blue shift in the SPR peak position to 430 nm indicating the formation of small particles. At 75 °C, the SPR peak become blues shifted at the beginning of the reaction indicating the formation of smaller particles compared to those synthesised at lower temperature. However, as the reaction time increased, the SPR peak position becomes red-shifted indicating increase in particle size. This observation is contrary to the result obtained at lower temperature in which the SPR peak position become blue-shifted as the reaction time increased. The formation of bigger particles at 75 °C as the reaction time increased is attributed to aggregation due to the degradation of some thermo-labile phytochemicals that are present in the plant extract under high temperature as the reaction time increased. The degradation of these phytochemicals led to insufficient passivation of the Ag-NPs as the reaction continued and hence aggregation. Similar observation had been reported by Ahmad *et al.*, for the biosynthesis of gold and silver NPs [8].

### 2.3. FTIR Analysis

**Figure 2:**
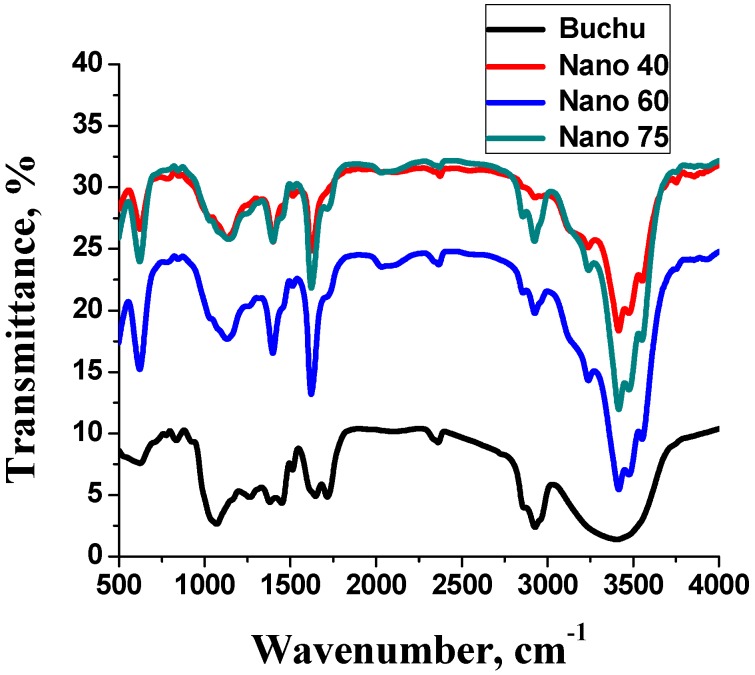
Fourier transform infra-red spectroscopy (FTIR) spectra of buchu ethanolic extract and as-synthesised silver nanoparticles (Ag-NPs) at different temperatures.

Figure 2 shows the FTIR spectra for the ethanolic extract and the as-synthesised Ag-NPs. The FTIR study of the extract and the Ag-NPs showed absorption bands corresponding to functional groups found in secondary metabolites. This indicated that secondary metabolites are involved in the synthesis of Ag-NPs. The prominent peaks in the extract were 3400, 2900 and 1100 cm^−1^. The prominent peaks in the silver nanoparticles were 396, 460, 2900 and 3400 cm^−1^. The broad peak in the crude at 3400 cm^−1^ is typical of a hydroxyl group from carbohydrates and amides. The peak at 2900 cm^−1^ indicates the presence of alkyl groups while 1100 cm^−1^ signifies the presence of C-O functional groups [9,10]. Phytochemical screening of the crude confirmed the presence of proteins, carbohydrates and flavonoids. Alcohols have been reported to facilitate the reduction of silver ions to silver nanoparticles while they are oxidised to carbonyl compounds [10]. Flavonoids are capable of releasing free reactive hydrogen during their keto-enol tautomeric transformations which can also facilitate the reduction of silver ions to silver nanoparticles [11]. Further analysis of the Ag-NPs spectra revealed that the first two bands at 396 cm^−1^ and 460 cm^−1^ originate from Ag-NPs ligand stretching vibrations that appear due to interaction of bio-molecules with the nanoparticle [12]. The absorption band at 3400 cm^−1^ indicates the presence of NH groups or OH bonded to a carbohydrate. This shows that the NH and OH groups among a host of other functional groups could be broadly responsible for the reduction of ionic silver to zero valent silver nanoparticles [11]. The peak at 2900 cm^−1^ is attributed to the alkane stretching band. The decrease in intensity between the extract and the nanoparticles indicates a realignment of C-H bonds of the organic compounds in the crude during the nanoparticle synthesis [9]. The peaks observed from FTIR analysis confirm the reduction and capping of the as-synthesised Ag-NPs by the phytochemicals present in the ethanolic extract.

### 2.4. TEM Analysis

**Figure 3:**
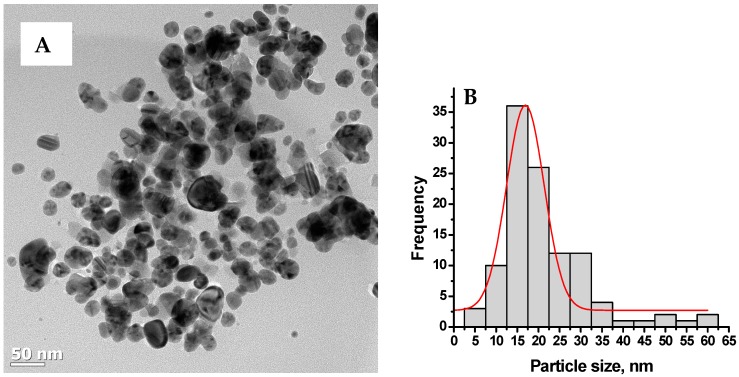
Typical transmission transform microscopy (TEM) images of Ag-NPs synthesised at 60 °C (**A**) and their size distribution (**B**).

Figure 3 shows the representative TEM image and size distribution of buchu ethanolic extracts synthesised Ag-NPs at 60 °C. The micrograph (Figure 3A) shows a mixture of small and bigger particles that are spherical and oblong in shapes. The size distribution curve (Figure 3B) shows that the particles are in the range 5–60 nm with average particle diameter of 19. 95 ± 7.76 nm.

### 2.5. Analgesic Activity

**Figure 4:**
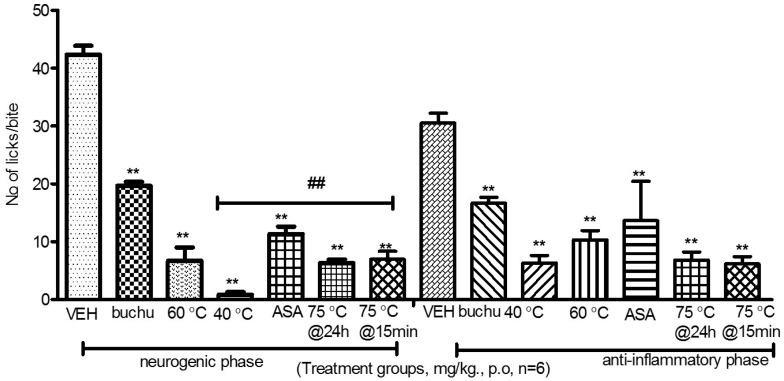
Effect of nanoparticles on paw licking in mice. ** *p* < 0.01 statistically lower than the vehicle. ## *p* < 0.01 statistically lower compared to buchu crude extract. VEH is the vehicle; ASA is Aspirin drug; 40 °C, 60 °C and 75 °C are the nanoparticles synthesized at 40 °C, 60 °C and 75 °C; 75 °C@15 min are nanoparticles synthesized at 75 °C harvested after 15 min; 75 °C@24 h are nanoparticles synthesized at 75 °C harvested after 24 h.

The results obtained from analgesic activity test show that all the as-synthesised Ag-NPs significantly reduced the number of paw licks at a dosage of 200 mg/kg. The inhibition values for all the as-synthesised Ag-NPs are between 73% and 98% (*p* < 0.05) for the neurogenic phase and between 55%–80% for the anti-inflammatory phase. The inhibition values for buchu crude are 55% and 45% for the neurogenic and the anti-inflammatory phase respectively. The inhibition values for the positive control (Aspirin) are 84% and 81% for the neurogenic and the anti-inflammatory phase respectively (*p* < 0.05). The Ag-NPs significantly reduced the number of paw licks, indicating that the as-synthesised Ag-NPs have the ability to inhibit the action of pain mediators (Figure 4).

Analysis of variance between the experimental groups showed that the inhibition values of the nanoparticles were significantly higher than those administered with buchu ethanolic extract alone and aspirin. This has been attributed to the synergistic effect between the plant secondary metabolites and Ag-NPs. Furthermore, the Ag-NPs significantly inhibited the effect of inflammation mediators such as prostaglandins and its derivatives that cause pain and inflammation more than both aspirin and the ethanolic extract [13]. The nanoparticles produced at 75 °C at 15 min reaction time had the smallest inhibition values for both the neurogenic and anti-inflammatory phase among the as-synthesised Ag-NPs. This has been attributed to the low activity of the plant extract at the beginning of the reaction. These results show that the combined effects of the plant extract and Ag-NPs are more effective than buchu extracts in the management of pain.

## 3. Materials and Methods

### 3.1. Plant Material and Preparation of Extract

Buchu plant material was obtained from King William’s Town in the Eastern Cape Province of South Africa and authenticated at Botany Department, Walter Sisulu University. The plant material (leaves) was cleaned, air dried at 20 °C–25 °C and prepared for solvent extraction using ethanol (95% *v*/*v*). The extract obtained was filtered and concentrated using a vacuum evaporator at 30 °C. The ethanolic extract was screened for secondary metabolites (tannins, flavonoids, saponins, steroids, terpenes, glycosides, proteins and alkaloids) using phytochemical screening methods as described by Harbone and Sofowora [14,15].

### 3.2. Synthesis of Silver Nanoparticles

Thirty grams of buchu ethanolic extract were dissolved in 100 mL of water and filtered to create a stock solution. From this stock solution 10 mL was taken and mixed with 10 mL of 0.1 M AgNO_3_. This mixture was diluted to 50 mL with distilled water and heated to 40 °C under continuous stirring. Aliquots were taken at different reaction time to monitor the growth of Ag-NPs. The experiment was repeated at different temperatures of 60 °C and 75 °C.

### 3.3. Characterisation

The reduction of Ag-NPs was monitored using UV 1650 PC-Shimadzu B UV-visible spectrophotometer (Shimadzu, Osaka, Japan). TEM analysis was carried out using a JEOL JEM 2100 (TEM) operated at 200 KV. Samples were mounted on a carbon coated grid. FTIR analysis was done by using a Perkin Elmer Spectrophotometer (Los Angeles, CA, USA).

### 3.4. Analgesic Activity

Forty two Swiss albino mice weighing 20–32 g of both sexes were obtained from the animal room at Walter Sisulu University. Room temperature was maintained at 24 °C while lighting was provided exclusively by daylight. All the animals were used according to the Walter Sisulu Ethical Clearance Committee (No. 0009/07) ethics. The formalin test was carried out to test for analgesic activity as described by Prabhu *et al.* with some modifications [16]. The mice were divided into seven groups as follows:

- Control group—0.9% *w*/*v* (normal saline)
- Standard group—100 mg/kg aspirin
- Group III—200 mg/kg ethanolic extract
- Group IV—200 mg/kg of nano particles produced at 40 °C
- Group V—200 mg/kg of nano particles produced at 60 °C
- Group VI—200 mg/kg of nano-particles at 75 °C (15 min reaction time)
- Group VII—200 mg/kg nanoparticles produced at 75 °C (24 h reaction time)

The control group received 0.9% NaCl (10 mL/kg), the standard group received aspirin, group III received buchu extract, and groups IV–VII received the nanoparticles. One hour (1 h) after treatment with the various drugs, animals were injected sub-plantarly with a 100 μL of 2.5% formalin solution (diluted in saline). Animals responded to formalin injection by licking the injured paw. The number of times the mice licked the paw was recorded during the first 5 min (neurogenic phase) and then 20 to 30 min (inflammatory phase) after formalin injection. % inhibition = (1 − (T/C)) × 100. T is the number of times treated mice licked the injured paw and C is the number of times control mice licked the treated paw.

## 4. Conclusions

Green synthesis of silver nanoparticles and its analgesic activities using buchu ethanolic extract is reported for the first time. The Ag-NPs showed significant inhibition of pain as compared to aspirin a standard analgesic drug at a dosage of 200 mg/kg. The high inhibition values obtained in the analgesic test for the as-synthesised Ag-NPs compared to the extract is attributed to the synergistic effect between the plant secondary metabolites and Ag-NPs. These analyses show that as-synthesised Ag-NPs can be used in the management of pain.

## Figures and Tables

**Table 1:** Phytochemical screening results.

Metabolite	Ethanolic Extract
Glycosides	+
Flavonoids	+
Alkaloids	+
Terpenes	+
Steroids	+
Tannins	+
Saponins	+
Proteins	+

+ Present.
